# Supplementary material for: XPF mediates 3’ flap processing for FEN1-independent Okazaki fragment maturation
Source: bioRxiv. 2025 Nov 1:2025.10.24.684453. Preprint. [Version 2] doi: 10.1101/2025.10.24.684453 (PMC12633238; doi:10.1101/2025.10.24.684453)
Supplement: Supplement 1 [file media-1.pdf]

Table S1: Random spore analysis of the SL phenotype of *rad1*Δ *rad27*Δ and suppression of the SL phenotype by *pol3* ITD

Genetic cross	Colony Number (YPD plates)	Colony Number (SC plates)	Observed ratio	Expected Ratio	Viability
*rad1*Δ::Trp1<br>×<br>*rad27*Δ::Ura3	103	*rad1*Δ *rad27*Δ<br>3	0.029	0.25	NO
*rad1*Δ::Trp1<br>×<br>*rad27*Δ::Ura3<br>*pol3* ITD::His3	154	*rad1*Δ *rad27*Δ *pol3* ITD<br>17	0.110	0.125	YES
*rad1*Δ::Trp1<br>×<br>*pol3* ITD::His3	645	*rad1*Δ *pol3* ITD<br>241	0.374	0.25	YES

Table S2: Random spore analysis of the SL phenotype of *mre11*Δ *rad27*Δ and suppression of the SL phenotype by *pol3* ITD

Genetic cross	Colony Number (YPD plates)	Colony Number (SC plates)	Observed ratio	Expected Ratio	Viability
*mre11*Δ::Trp1<br>×<br>*rad27*Δ::Ura3	961	*mre11*Δ *rad27*Δ<br>6	0.006	0.25	NO
*mre11*Δ::Trp1<br>×<br>*rad27*Δ::Ura3<br>*pol3* ITD::His3	768	*mre11*Δ *rad27*Δ *pol3* ITD<br>3	0.004	0.125	NO
*mre11*Δ::Trp1<br>×<br>*pol3* ITD::His3	606	*mre11*Δ *pol3* ITD<br>174	0.287	0.25	YES

Table S3: Random spore analysis of the SL phenotype of *mus81*Δ *rad27*Δ and suppression of the SL phenotype by *pol3* ITD

Genetic cross	Colony Number (YPD plates)	Colony Number (SC plates)	Observed ratio	Expected Ratio	Viability
*mus81*Δ::Trp1<br>×<br>*rad27*Δ::Ura3	81	*mus81*Δ *rad27*Δ<br>2	0.025	0.25	NO
*mus81*Δ::Trp1<br>×<br>*rad27*Δ::Ura3<br>*pol3* ITD::His3	400	*mus81*Δ *rad27*Δ *pol3* ITD<br>61	0.153	0.125	YES
*mus81*Δ::Trp1<br>×<br>*pol3* ITD::His3	81	*mus81*Δ *pol3* ITD<br>18	0.222	0.25	YES

Table S4: Random spore analysis of the SL phenotype of *sae2*Δ *rad27*Δ and suppression of the SL phenotype by *pol3* ITD

Genetic cross	Colony Number (YPD plates)	Colony Number (SC plates)	Observed ratio	Expected Ratio	Viability
*sae2*Δ::Trp1 × *rad27*Δ::Ura3		*sae2*Δ *rad27*Δ		0.25	NO
*sae2*Δ::Trp1 × *rad27*Δ::Ura3 *pol3* ITD::His3	530	*sae2*Δ *rad27*Δ *pol3* ITD 3	0.006	0.125	NO
*sae2*Δ::Trp1 × *pol3* ITD::His3		*sae2*Δ *pol3* ITD		0.25	

Table S5: Random spore analysis of the SL phenotype of *sgs1*Δ *rad27*Δ and suppression of the SL phenotype by *pol3* ITD

Genetic cross	Colony Number (YPD plates)	Colony Number (SC plates)	Observed ratio	Expected Ratio	Viability
*sgs1*Δ::Trp1<br>×<br>*rad27*Δ::Ura3	68	*sgs1*Δ *rad27*Δ<br>0	0	0.25	NO
*sgs1*Δ::Trp1<br>×<br>*rad27*Δ::Ura3<br>*pol3* ITD::His3	38	*sgs1*Δ *rad27*Δ *pol3* ITD<br>5	0.132	0.125	YES
*sgs1*Δ::Trp1<br>×<br>*pol3* ITD::His3	68	*sgs1*Δ *pol3* ITD<br>16	0.235	0.25	YES

Table S6: Random spore analysis of the SL phenotype of *srs2*Δ *rad27*Δ and suppression of the SL phenotype by *pol3* ITD

Genetic cross	Colony Number (YPD plates)	Colony Number (SC plates)	Observed ratio	Expected Ratio	Viability
*srs2*Δ::hph × *rad27*Δ::Leu2	115	*srs2*Δ *rad27*Δ 0	0	0.25	NO
*srs2*Δ::hph × *rad27*Δ::Leu2 *pol3* ITD::His3	223	*srs2*Δ *rad27*Δ *pol3* ITD 7	0.031	0.125	NO
*srs2*Δ::hph × *pol3* ITD::His3	1226	*srs2*Δ *pol3* ITD 254	0.207	0.25	YES

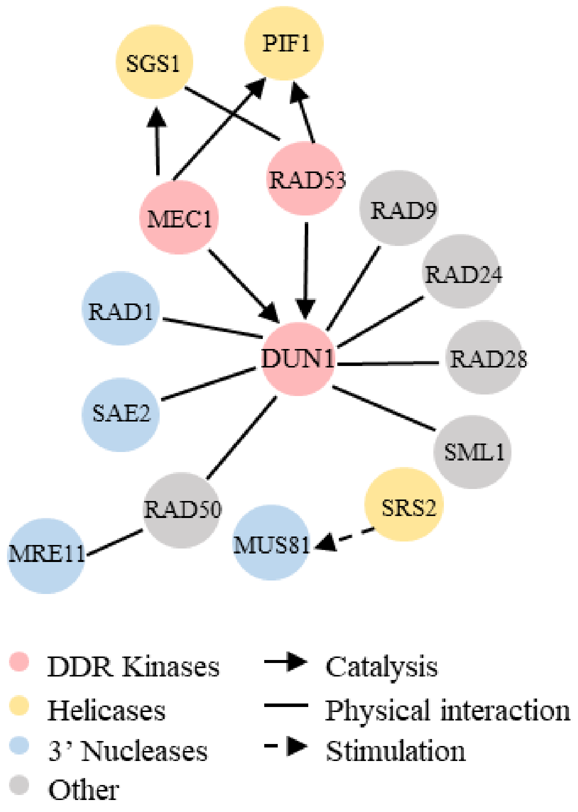

Supplementary Figure S1: **Physical interactions of DNA repair proteins with the Mec1-Rad53-Dun1 axis.** Saccharomyces Genome Database (SGD) was surveyed for DUN1 interaction helicases and nucleases.

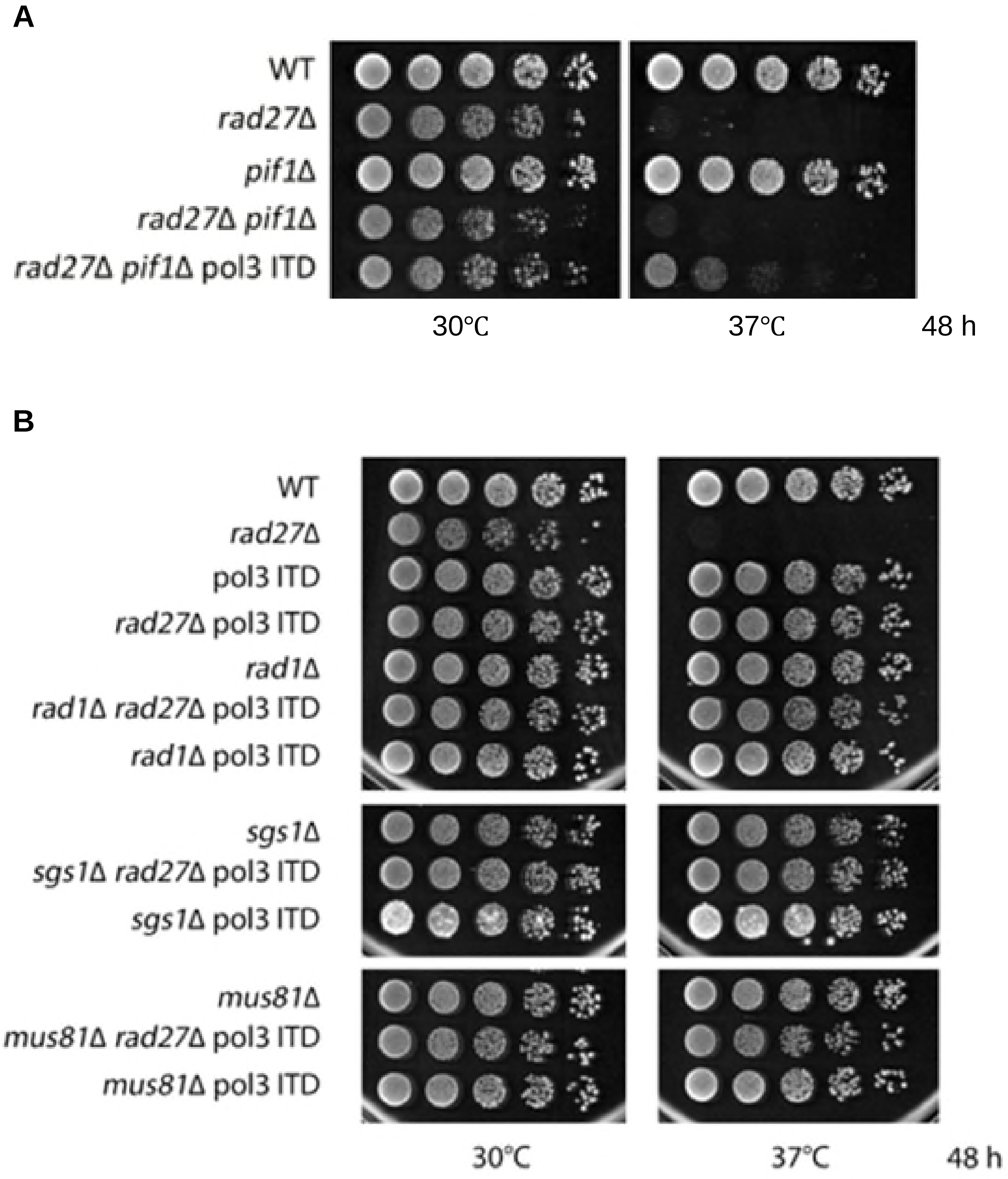

Supplementary Figure S2: Spot assays to verify the viability of yeast cells. (A) Spot assays on WT, *rad27*Δ, *pif1Δ*, *rad27*Δ *pif1Δ, or rad27*Δ *pif1Δ* pol3-ITD at 30℃ (optimal temperature) or 37℃ (restrictive temperature). **(B)** Spot assays on WT, *rad27*Δ, *rad1Δ*, *sgs1Δ, mus81Δ, rad27*Δ *rad1Δ, rad27*Δ *sgs1Δ, rad27*Δ *mus811Δ, or rad27*Δ *rad1Δ pol3-ITD, rad27*Δ *sgs1Δ pol3-ITD, rad27*Δ *mus811Δ pol3-ITD* at 30℃ or 37℃.

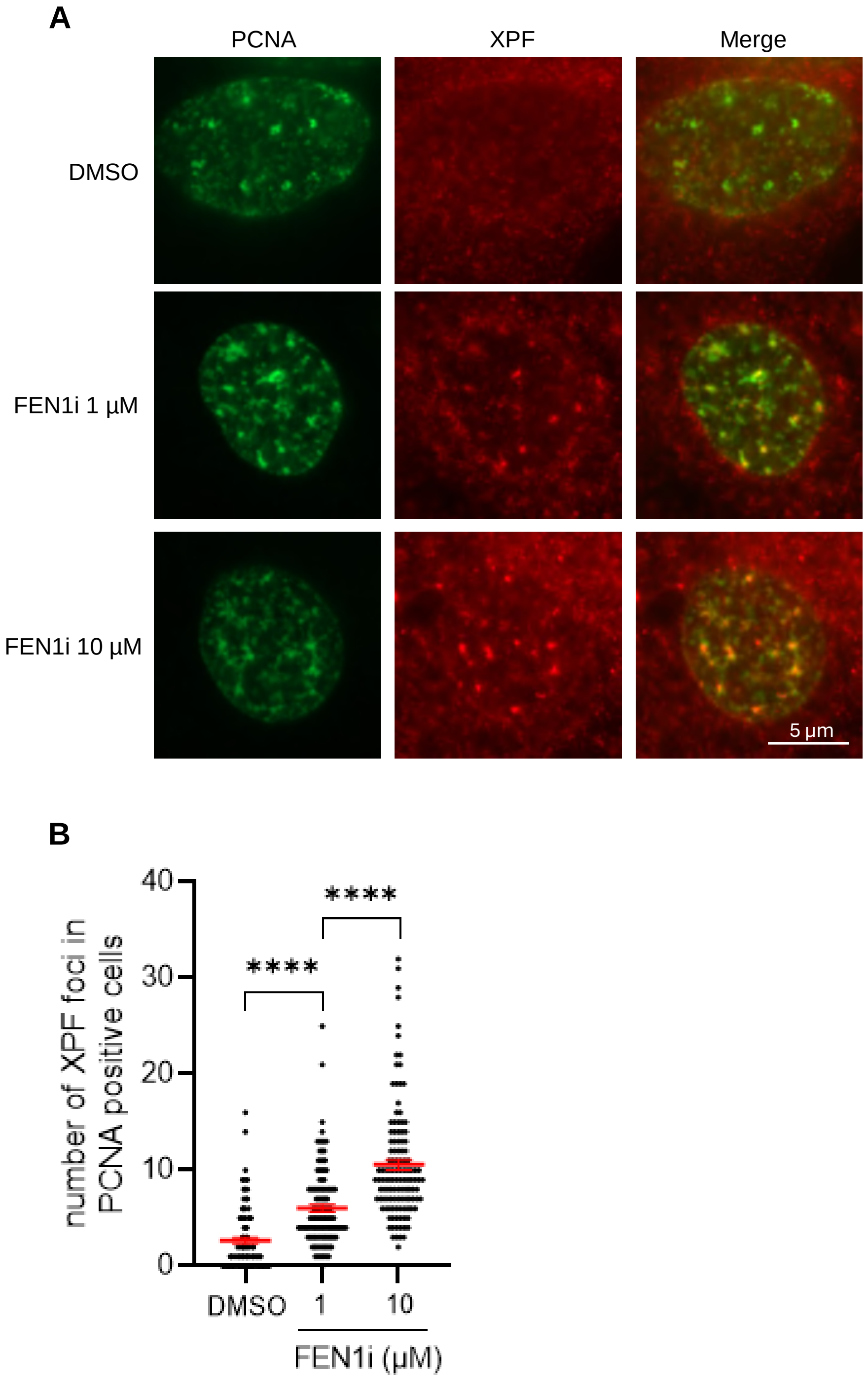

Supplementary Figure S3: co-IF staining of PCNA and XPF in MEFs in the absence or presence of FEN1i. MEFs were treated with DMSO or FEN1i (1 µM or 10 µM LNT-1) for 16hr. Co-IF staining of PCNA and XPF was carried out. **(A)** Representative microscope images of XPF-PCNA co-IF staining in MEF cells treated with DMSO or FEN1i; **(B)** Quantification of the XPF-PCNA co-localized foci number per cell. Data represent mean ± SEM from ≥100 cells per condition. **** $p < 0.0001$. P values were calculated with the Student t-test.

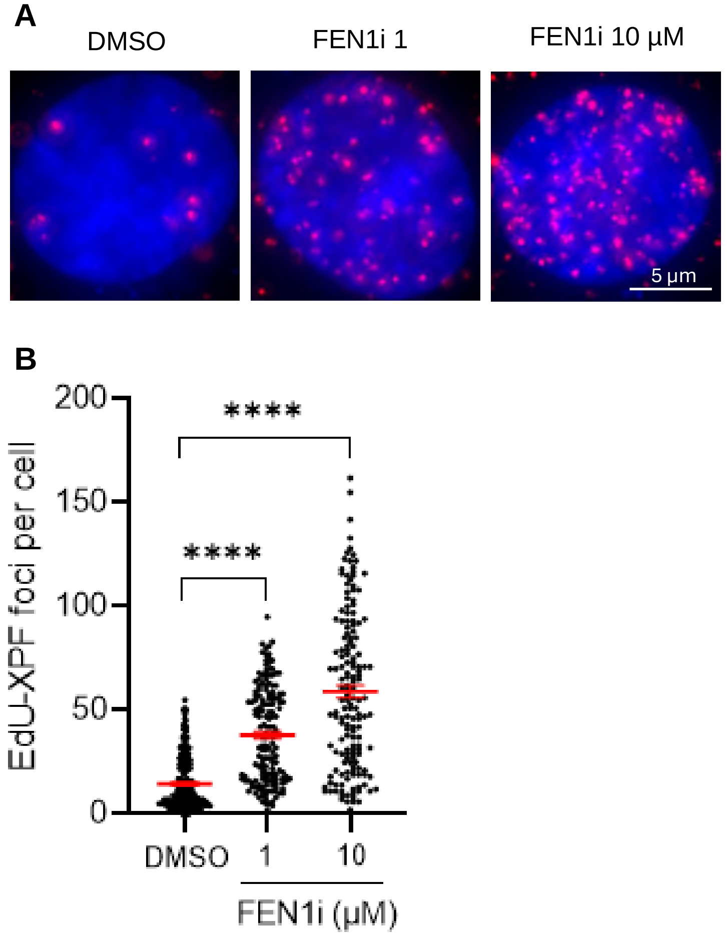

Supplementary Figure S4: The EdU-XPF PLA assay shows XPF recruitment to nascent DNA in MDA-MB-231 cells. **(A)** Representative microscope images of PLA performed in MDA-MB-231 cells treated with DMSO or FEN1i; **(B)** Quantification of the EdU-XPF PLA foci number in MDA-MB-231 cells. Cells were treated with DMSO or FEN1i (1and 10 µM) for 16hr and labeled with10 µM EdU before harvest. Data represent mean ± SEM from ≥100 cells per condition. ****P < 0.0001. P value from Student t-test.

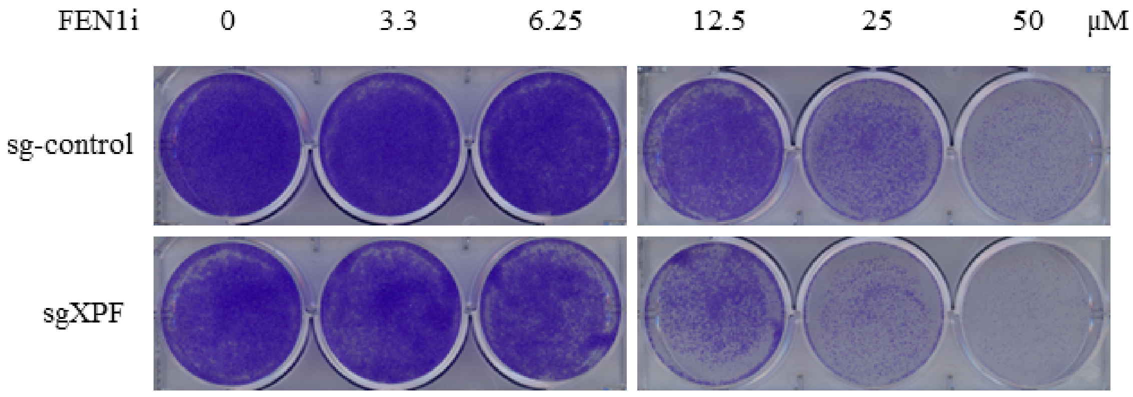

Supplementary Figure S5: Cell viability assay in WT (sg-control) or ERCC4-/- (sgXPF) MDA-MB-231 cells. WT or ERCC4-/- cells were cultured in DMEM containing varying concentrations of FEN1i (LNT-1) for 10 days. Viable cells were stained with Crystal-violet solution.
